# Identification of novel genera and subcluster classifications for mycobacteriophages

**DOI:** 10.20517/mrr.2023.17

**Published:** 2023-06-15

**Authors:** Laura M. O’Connell, Colin Buttimer, Francesca Bottacini, Aidan Coffey, Jim M. O’Mahony

**Affiliations:** ^1^Munster Technological University, Cork T12 P928, Ireland.; ^2^APC Microbiome Ireland, Biosciences Research Institute, University College, Cork T12 YT20, Ireland.

**Keywords:** Mycobacteriophage, taxonomy, ICTV, actinobacteriophage database, novel genera, novel subclusters

## Abstract

**Aim:** To identify novel genera amongst mycobacteriophages (MP) and verify a hypothesised correlation between the taxonomy set by the International Committee on Taxonomy of Viruses (ICTV) and the National Centre for Biotechnology Information (NCBI) with that of the Actinobacteriophage Database, which may help formalise subcluster assignment.

**Methods:** A dataset of 721 MP genomes was analysed using VIRIDIC, a nucleotide alignment-based software that predicts genus assignments. Potentially novel genera were analysed using Gegenees and VICTOR, respectively. These genera were then compared to the subclusters assigned by the Actinobacteriophage Database to verify a hypothesis that one genus can be assigned to one subcluster (i.e., the genus-subcluster hypothesis).

**Results:** Initially, when comparing the current genus classifications of the 721 MP dataset to the Actinobacteriophage database subcluster assignments, 83.3% of subclusters supported the genus-subcluster hypothesis. Following the sequential VIRIDIC, Gegenees and VICTOR analyses, a total of 20 novel genera were identified based on a ≥ 70% and ~ 50% similarity threshold for VIRIDIC and Gegenees, respectively, and a monophyletic nature in the VICTOR output. Interestingly, these criteria also appear to support the creation of 13 novel subclusters, which would increase the support for the genus-subcluster hypothesis to 97.6%.

**Conclusion:** The link between genus and subcluster classifications appears robust, as most subclusters can be assigned a single genus and vice versa. By relating the taxonomic and clustering classification systems, they can be easily kept up to date to best reflect MP diversity, which could aid the rapid selection of related (or diverse) phages for research, therapeutic and diagnostic purposes.

## INTRODUCTION

The frequency of antibiotic resistance (AR) is increasing at a worrying rate. There has been an increase in resistant nontuberculosis mycobacteria (NTM) infections, demonstrating varying levels of AR^[1,2]^. Although NTM are typically opportunistic pathogens, treatment failure may lead to stubborn colonisation^[2]^. For example, *Mycobacterium avium* sbsp. *paratuberculosis* (MAP) causes chronic gastroenteritis (i.e., Johne’s disease) in ruminant animals^[3]^. In order to address the challenges of AR-related mycobacterial infections, alternatives to traditional antibiotic regimens are actively being explored. One alternative is mycobacteriophage (MP) therapy^[4]^. Recently, human case studies involving effective MP therapy were described, the first being the successful treatment of a young cystic fibrosis patient with a chronic AR *Mycobacterium abscessuss* pulmonary infection^[5,6]^. As the number of isolated and sequenced phages increases, greater opportunity to create robust MP therapy will arise, either through identifying or engineering phages capable of infecting NTM.

As the genomics era progresses, sequencing and annotation of phage genomes have become as routine and vital as phenotypic characterisation (e.g., host range, burst size, and adsorption assays). The ever-increasing volume of available genomic information and sequence analysis software allows for more in-depth *in silico* analyses that may provide valuable insights regarding the potential functionality and taxonomy of phages, as suggested by Lawrence *et al.*^[7]^. By exploring the genomic data further, it may be possible to identify other characteristics (e.g., host range, pH tolerance, heat tolerance) shared by phages belonging to the same taxonomic groups (e.g., those belonging to the same genus, subfamily, or family group) that may greatly aid the design of phage therapies and diagnostics. However, assumptions made while characterising a new isolate based on their supposed taxonomy can only be trusted if phage taxonomy is well maintained^[8,9]^.

The latest software to be introduced for identifying phage taxonomic relationships is VIRIDIC, which calculates intergenomic similarities between viral genomes in a pairwise manner, as well as their length ratio and the aligned genome fraction^[10]^. The output of the algorithm includes a hierarchical heatmap of the similarity scores, which places the most similar genomes together. This heatmap is accompanied by a cluster table that indicates genus- and species-level relationships based on pre-set thresholds of genomic similarity^[10]^. The default settings of VIRIDIC are set to identify genome groups based on the latest taxonomic demarcations, i.e., genus threshold of ≥ 70% and species threshold of ≥ 95%^[10,11]^. During its development, it was noted that VIRIDIC produced results that most closely supported those of the traditional BLASTN algorithm, while outperforming other bioinformatic tools with regard to estimating the relatedness between more distally related genomes^[10]^. The use of VIRIDIC to identify unknown or outdated phage taxonomy has become commonplace (e.g., classifying phages targeting *Pseudomonas*, *Salmonella*, *Vibrio*, and *Bacillus*) since its development in 2020, as systems move toward sequence-based classifications, as predicted by Lawrence *et al.* in 2002^[7,10,12-17]^. With this in mind, the existing taxonomy of publicly available MP was interrogated to determine whether the current classifications remain accurate or require revisions. Thus far, global efforts have isolated almost 12,000 MP, and over 2,100 have been fully sequenced, largely as part of the SEA-PHAGES program. The program initially involved undergrad students undertaking massive screening efforts to identify novel MP; however, the scope has broadened to include phages targeting other bacteria^[18,19]^. Notably, the majority of MP identified thus far have been isolated using a single host strain, *Mycobacterium smegmatis* mc^2^ 155, which creates a degree of bias with regard to the types of phages isolated and characteristics such as host range and this bias may obscure the true diversity of MP^[20]^.

In this investigation, VIRIDIC was used to group MP into genera. The subsequent VIRIDIC-defined taxonomy was compared to the existing taxonomy within the National Centre for Biotechnology Information (NCBI) database and the International Committee on Taxonomy of Viruses (ICTV) Master Species List. The potentially novel genera suggested by VIRIDIC were further supported by proteomic analyses performed using Gegenees and VICTOR. The purpose of Gegenees is to fragment complete genomes to the default set length and search (using tBLASTx) the appropriate BLAST database for “seeds” of each fragment against the other genomes. These results can then be used to infer phylogenetic distances^[21,22]^. VICTOR visualises phylogeny based on comparisons of genome or proteome sequences to generate dendrograms extrapolated from the genome-BLAST distance phylogeny method with branch support^[23]^. If the novel VIRIDIC predicted genera are supported, it was hypothesised that the Gegenees output would mirror the VIRIDIC alignments, although it should be noted that comparing DNA-based to proteomic-based similarity values is complicated by complex evolutionary patterns, genetic exchange events and the mosaic nature of MP^[24]^. Each genus was also anticipated to be represented by a monophyletic branch (or clade) within the VICTOR-generated dendrogram.

The VIRIDIC-assigned groups were also compared to the existing cluster/subcluster assignments of the phages to begin investigating a hypothesis that was generated during the initial curation of the MP into their respective subclusters. Essentially, this study proposes a link between subclusters and genera. The cluster-based classification system was initially established to aid the organisation of the outputs from the SEA-PHAGES program and later broadened into a large public database featuring phages targeting a variety of hosts, i.e., the Actinobacteriophage Database (https://phagesdb.org/). Originally, cluster assignment required all members to share 50% nucleotide similarity of their total genomes^[18,19]^, but now requires that phages share 35% of their gene content based on a bioinformatic pipeline involving the Phamerator program, which assigns genes into groups of related sequences^[19,25]^. This cluster demarcation (≥ 50% nucleotide similarity) was noted to have been the minimum similarity required for genus assignment until recently^[12]^. Therefore, one would expect each cluster to consist of a single genus. However, if the latest genus demarcation requires a minimum of 70 % genome similarity^[10,12]^, it can be hypothesised that more than one genus may exist within a single cluster using this threshold. As subcluster division within clusters is largely based on subgroups of genomes having evidently higher nucleotide similarities to each other than the cluster as a whole, it may be possible for subclusters to reflect the most up-to-date demarcation of genus (i.e., > 70% similarity; ^[12,18,20,26]^). Therefore, similar sequential analyses using VIRIDIC, Gegenees, and VICTOR could likely identify novel subcluster groups. Should this hypothesis prove correct, it could lead to a formalisation of the criteria for subcluster creation, which is currently arbitrarily based on “recognisable divisions” within comparisons of average nucleotide identity in each cluster (Hatfull, 2022). It is also widely noted that subcluster thresholds vary between clusters, including those of phages that infect bacteria other than mycobacteria^[27]^. Therefore, the overall purpose of this analysis was to not only identify novel genera, but to establish a more consistent method of subcluster assignment amongst MP.

## METHODS

### Initial selection of MP genomes from established bioinformatic databases.

When this research began, 2,096 MP genomes had been fully sequenced, and the majority of this data was available through Genbank. In order to generate a more manageable dataset and to create a “snapshot” of MP taxonomy, the initial dataset of MP genomes was restricted to those available from the RefSeq database, thereby reducing the data set to 752 genomes (https://www.ncbi.nlm.nih.gov/labs/virus/vssi/#/virus?SeqType_s=Genome, accessed 14 December 2021). The genomes were then manually organised into their respective clusters, which were assembled from publications associated with the characterisation of MP and the Actinobacteriophage Database (https://phagesdb.org/hosts/genera/1/?sequenced=True, accessed 14 December 2021). At this point, it was decided to introduce exclusion criteria to remove small clusters of genomes as the scope of this study is quite broad, and it was hypothesised that novel groupings would be more apparent in larger clusters due to the larger sample size. The criteria for exclusion from the study were: (1) MP that could not be assigned to a cluster based on the literature/database information; (2) clusters represented by ≤ 3 MP genomes (considered underrepresented); and (3) MP described as singletons (which lack sufficient nucleotide identity and/or shared gene content to be clustered with known phages; ^[28]^). Following these criteria, 15 groupings (comprised of 30 genomes total) of MP genomes were removed from the dataset prior to VIRIDIC analysis, as detailed in the Results. To ensure the removal of these 30 genomes was not likely to influence the outcome of the analyses, each genome was analysed with BLASTN (https://blast.ncbi.nlm.nih.gov/Blast.cgi?PROGRAM=blastn&PAGE_TYPE=BlastSearch&LINK_LOC=blasthome)to verify that they were not closely related to the remaining clusters.

### Manual curation of the taxonomic information related to the MP dataset.

The complete taxonomy of the shortlisted MP (721 genomes) from the RefSeq database was obtained from the NCBI (https://www.ncbi.nlm.nih.gov/; accessed 10-14 December 2022) and later cross-referenced with the most up-to-date information available from the (ICTV) Master Species List for conformational purposes (https://talk.ictvonline.org/files/master-species-lists/m/msl/12314, accessed 10 December 2022).

### Genomic analysis of the MP genomes using VIRIDIC and comparison of the VIRIDIC predicted taxonomy with the currently accepted taxonomic and subcluster classifications to identify novel groups.

**Figure 1:**
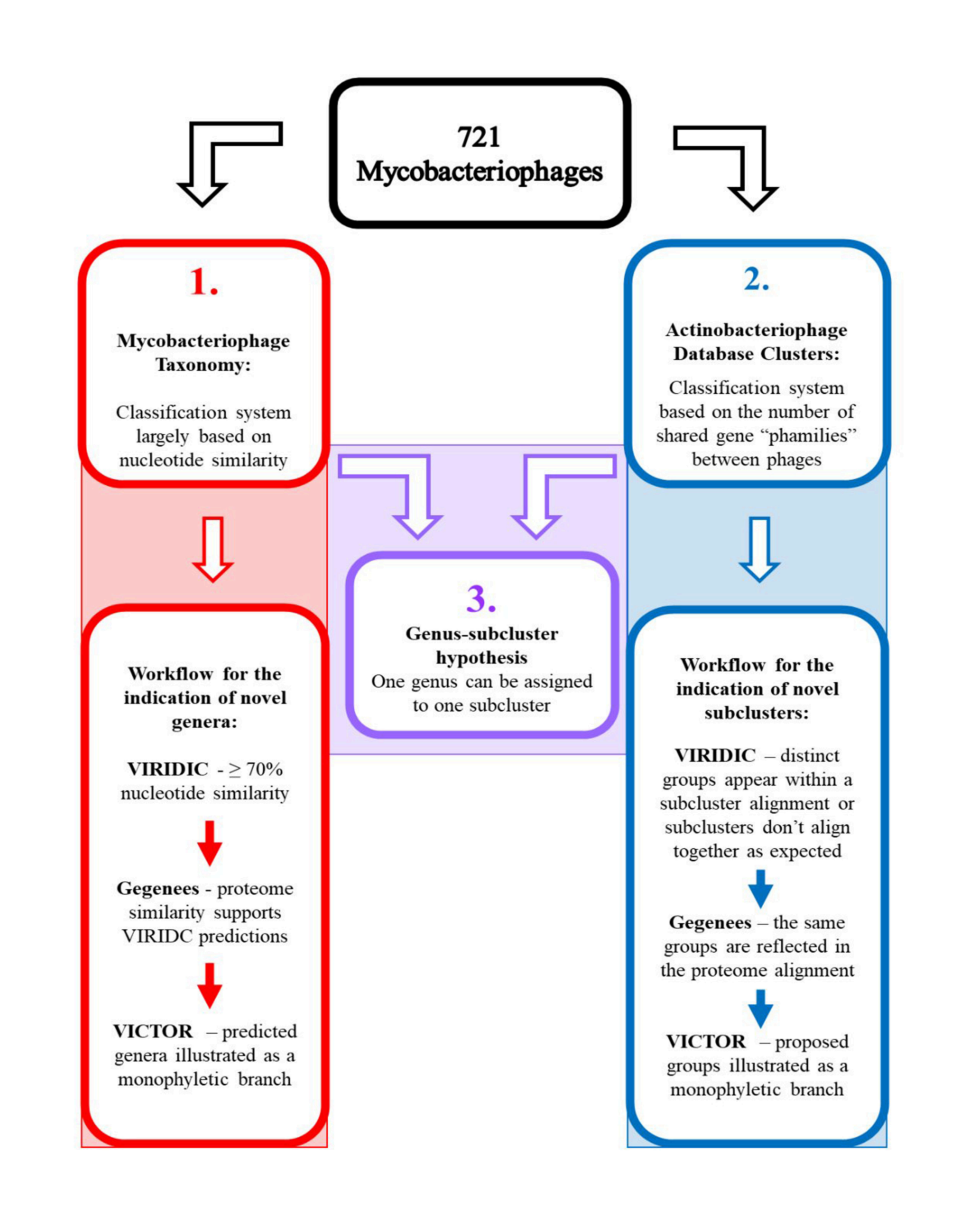
Flow chart illustrating the two classification systems. A summary of the identification of novel genera within the workflow for identifying novel taxonomy is presented in red and based on the proposed roadmap to genome-based phage taxonomy as outlined by Turner *et al.* (2021)^[12]^. The workflow for the recognition of novel subclusters within the Actinobacteriophage Database classification system is presented in blue. In the centre is the proposed overlap between the two systems (purple), which proposes that a single genus can be assigned to a single subcluster.

The whole genome sequences of the MP associated with each cluster were curated in FASTA format for the VIRIDIC analyses. The FASTA files were obtained from NCBI Virus (https://www.ncbi.nlm.nih.gov/labs/virus/vssi/#/virus?SeqType_s=Genome&SourceDB_s=RefSeq). The input format for VIRIDIC requires that all the genomic FASTA sequences included in the analysis are compiled into a single file. Once a file had been created for each cluster, they were individually inputted into the VIRIDIC web portal (http://rhea.icbm.uni-oldenburg.de/VIRIDIC/; ^[10]^). The resulting heatmaps and predicted genus groupings were compared with the current taxonomy for each cluster to identify potentially novel genera. The VIRIDIC outputs were also compared to the Actinobacteriophage database assigned subclusters to verify the genus-subcluster hypothesis and indicate where the creation of novel subclusters may be beneficial. Clusters that featured genera and/or subclusters that appeared novel were selected for further proteomic analysis using Gegenees and VICTOR, as outlined below [Figure 1].

### Proteomic analysis using Gegenees to provide support for the creation of novel genera and subclusters.

The MP genomes belonging to the VIRIDIC-assigned genera of interest were entered into Gegenees for TBLASTX (i.e., proteome) analysis using the default settings of a 200 bp fragmentation and a 100 bp step-size^[21]^. The fragmented approach is used alongside a multithread BLAST control engine to provide higher resolution of alignments^[21]^. The resulting values (presented in heatmap format) are the average BLAST scores expressed as a percentage of the score each genome would obtain when BLASTed against itself (i.e., 100 % identity; ^[21,22]^). The resulting heatmaps were expected to somewhat reflect the VIRIDIC heatmaps if the existence of the genera and subclusters were supported at a proteomic level^[10,21]^.

### Examination of amino acid-based phylogeny using VICTOR to illustrate the monophyletic nature of the proposed genera and subclusters.

Phylogenetic dendrograms based on the amino acid content of the genomes belonging to the proposed genera were created using VICTOR to provide additional evidence for their creation. VICTOR compares genome or proteome sequences to generate dendrograms extrapolated from the genome-BLAST distance phylogeny method with branch support^[23]^. The most highly supported trees (as calculated by the VICTOR server) were closely examined to determine whether the proposed genera were represented on a single branch, which is the expected presentation based on the roadmap to genome-based taxonomy proposed by Turner *et al*.^[12]^.

### Data presentation.

The heatmaps generated by VIRIDIC and Gegenees were created and/or edited using Microsoft Office Excel 365 (version 2203, Microsoft Corporation, Redmond, Washington, USA). “Conditional formatting” was used to introduce a colour scale to the VIRIDIC outputs, ranging from red (lowest score) to green (highest score). This type of conditional formatting is automatically applied by Gegenees when the software produces the alignments, so the heatmap outputs were simply exported as .html files and copied into Excel for formatting purposes. Specific ranges of similarity scores for each figure are indicated in the figure legends. At least one established genus was included in each analysis for comparative purposes, i.e., a “positive control” for how a genus should look in each output. Novel genera are indicated in bold black squares. The phylogenetic trees produced by VICTOR and ViPTree were digitally captured and edited in Microsoft PowerPoint (version 2203, Microsoft Corporation, Redmond, Washington, USA). The monophyletic branches representing genus groups are indicated with blue boxes and the proposed nomenclature is entered into each box. The subcluster groups are indicated in coloured boxes, while proposed subclusters are highlighted in red squares.

## RESULTS

### The initial selection of MP genomes from established bioinformatic databases

As outlined in the Materials and Methods, initial shortlisting of the Genbank genomes involved limiting the scope of the study to those included in the RefSeq database, which totalled 752 MP. The MP genomes were then organised into their respective clusters to identify additional phage that could be removed from this stage of the analysis based on whether the cluster appeared to be underrepresented, as described in the Materials and Methods [Table 1].

**Table 1:** The MP clusters represented in the RefSeq dataset, based on the information available on the Actinobacteriophage Database (https://phagesdb.org/hosts/genera/1/?sequenced=True) and/or literature searches

**Cluster**	**Number of Phage**	**Number of Subclusters**
A	256	19
B	63	12
C	31	2
D	5	2
E	25	0
F	157	5
G	18	5
H	6	2
I	6	2
J	11	0
K	66	7
L	22	4
M	7	2
N	19	0
O	5	0
P	24	6
Q	1	0
R	2	0
S	2	0
T	2	0
U	1	0
V	1	0
X	1	0
Y	2	0
Z	2	0
AA	1	0
AB	2	0
AC	3	0
AD	1	0
Singletons	6	n/a
Unknown	3	n/a

The total number of phage MP in each cluster as well as the total number of subclusters represented within the cluster are also indicated.

There were three phages that were not yet entered into the Actinobacteriophage Database, and their associated publications did not indicate their cluster or taxonomy, so these MP were not included in the initial analyses. Singleton phage and clusters represented by a single RefSeq genome (clusters Q, U, V, X, AA, and AD) were also not included due to their apparent underrepresentation in the dataset (> three genomes), as explained in the Materials and Methods. For this same reason, seven other clusters were removed - R, S, T, Y, Z, AB, and AC [Table 1]. BLASTN results indicated that the inter-cluster similarity of the removed clusters to those collated for the analysis is minimal and bears little to no influence on the results presented. By eliminating these clusters, the resulting dataset comprised 721 MP genomes grouped within 16 clusters (A-P), each represented by at least five genomes.

### Identification of novel genera and subcluster assignments by comparison of the current NCBI/ICTV taxonomy and Actinobacteriophage database subclusters with the VIRIDIC outputs

Following the taxonomic analysis summarised in Figure 1 in the Materials and Methods, it was determined that there were 20 potentially novel genera within clusters A, J and K, while the analysis of cluster G suggested that a single genus (and its respective subcluster) classification may not be strictly necessary. Regarding the proposed genus-subcluster hypothesis, it was noted that 83.3% of subclusters included in the dataset support the proposed relationship between genus and subcluster proposed in the Introduction. It is likely, based on the identification of 20 novel genera, that a greater percentage of agreement could be obtained if some of the remaining 16.7% of subclusters were reorganised into smaller subclusters to reflect the proposed genera while changing as few of the existing subclusters as possible to avoid confusion. In total, thirteen novel subcluster assignments were deemed robust enough based on the VIRIDIC, Gegenees and VICTOR analyses to be proposed in the following sections, and their creation increases the support for the genus-subcluster hypothesis from 83.3% to 97.6% when the 20 novel genera are also considered. The data regarding proposed changes to MP classifications are presented below in alphabetical order based on cluster. In each case, the VIRIDIC results are presented, followed by the Gegenees analyses and the phylogenetic trees to provide evidence for the existence of the novel genera. Subsequently, the queried subclusters are indicated within the VIRIDIC alignments and dendrograms to highlight anomalies with current subcluster assignments that may be clarified by the creation of more subclusters (or the removal of one, in the case of cluster G).

#### Cluster A - Ten novel genera and two novel subclusters

**Figure 2:**
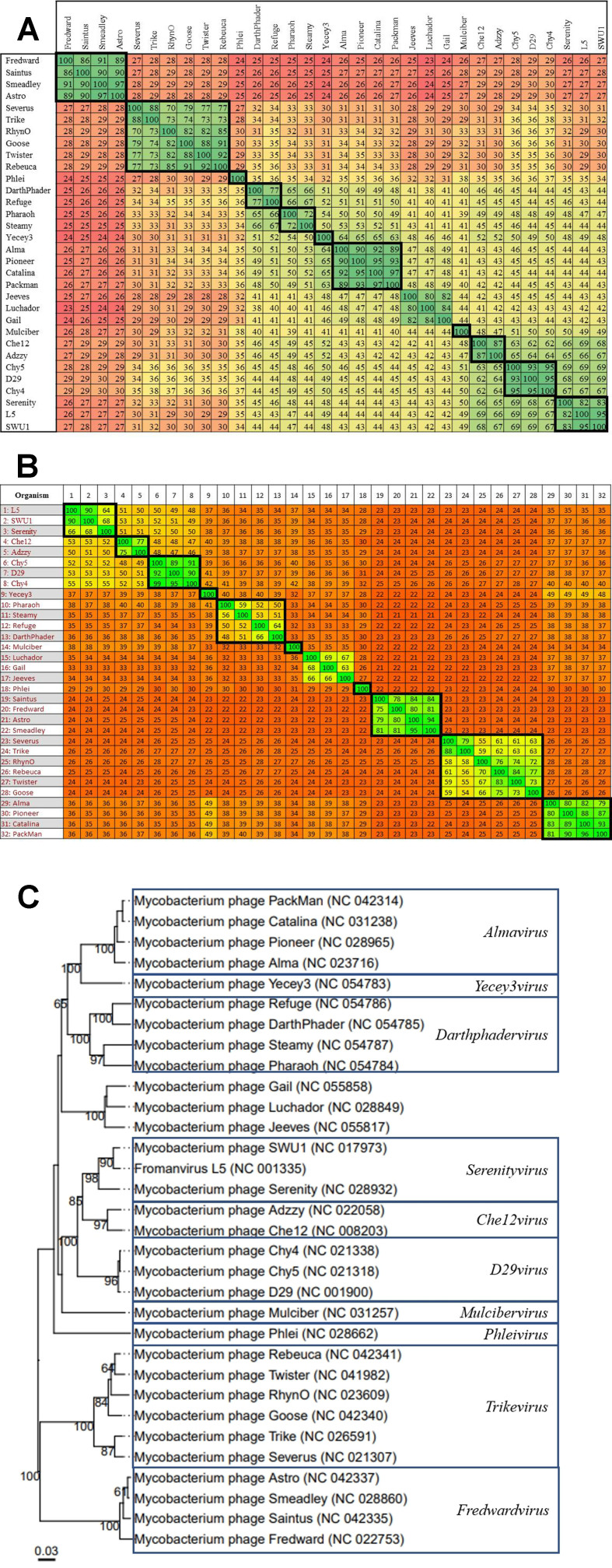
The supporting evidence for 10 novel genera within cluster A. The 256 MP dataset for this cluster was reduced to the genomes relevant for the description of the novel genera for legibility purposes. Novel genera are indicated in bold squares. The genus *Luchadorvirus* is included in each analysis to act as a control genus. (A) VIRIDIC analysis of the novel genera. The similarity values range from 24% to 100% nucleotide similarity. Based on a ≥ 70% nucleotide similarity threshold, 11 novel groups and one previously established group are observable; (B) Gegenees analysis of the novel cluster A genera. The proteome similarity values range from 21% to 100%. Most of the novel genera are clearly reflected in this analysis; however considering a ≥ 50% proteome similarity boundary, two of the VIRIDIC-identified genera can be combined and a total of 10 novel genera are observable; (C) VICTOR-generated dendrogram of the novel genera. The 10 novel genera are clearly represented as monophyletic clades that are similar to the “control” clade, i.e., the established genus *Luchadorvirus*.

The total number of genomes included in the dataset that belong to cluster A is 256. These are organised into 19 subclusters based on the existing Actinobacteriophage database classifications. There is a large number of MP assigned to the genus *Fromanvirus* [Supplementary Table 1]. The MP Froman belongs to subcluster A1, which VIRIDIC groups into a single genus, so this genus shall be considered the true *Fromanvirus* genus. Amongst the remaining “*Fromanviruses*”, initially, 11 novel genera were identified based on a threshold of ≥ 70% nucleotide similarity as illustrated in the VIRIDIC output [Figure 2A]. However, following inspection of the proteomic analysis performed with Gegenees, it appears that the best resolution of genera could be obtained based on a ≥ 50% proteome similarity threshold, which reduces the number of novel genera to ten [Figure 2B]. The ten genera were further supported by the VICTOR output, as each demonstrated a monophyletic nature similar to the genus *Luchadorvirus* which was included for comparative purposes [Figure 2C]. The suggested nomenclature for the novel groups is indicated in Figure 2C. Seven of these genera provide additional support to the proposed genus-subcluster relationship, with subclusters A8 and A10-13 each assigned to a single genus. The exceptions were subclusters A9 and A2. Of the five A9 MP included in the analysis, phage Yecey3 was assigned to a different genus (*Yecey3virus*) compared to the remaining four (*Almavirus*). Meanwhile, the eight A2 viruses were grouped into three genera (*Adzzyvirus*, *D29virus*, and *Serenityvirus*; Figure 2C).

**Figure 3:**
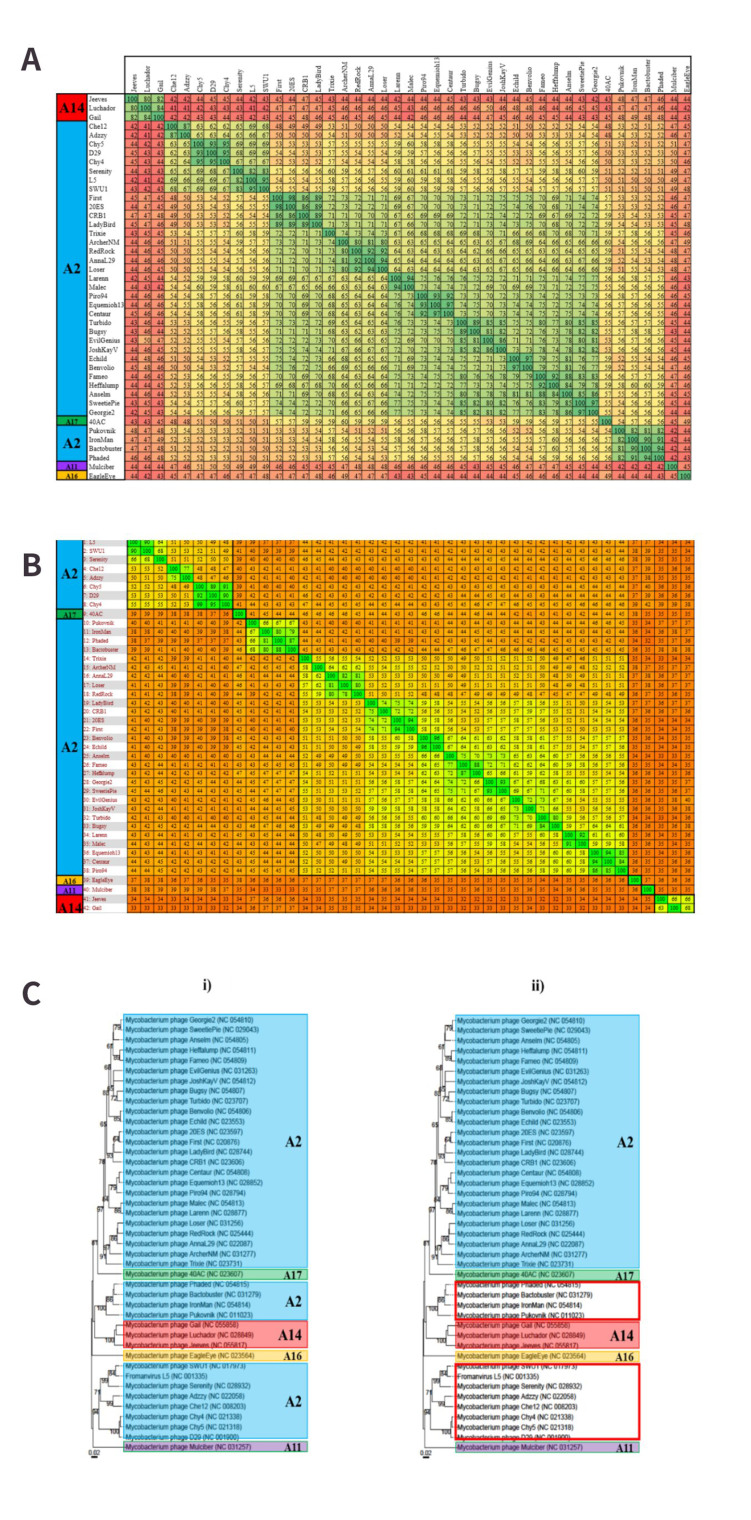
The creation of additional subclusters within cluster A. Novel groups are indicated in bold squares. (A) VIRIDIC analysis of the queried subclusters. The similarity values range from 41% to 100 % nucleotide similarity. Based on a ~ 50% nucleotide similarity threshold, three groups comprised of A2 phage can be observed; (B) Gegenees analysis of the queried subclusters. Considering a ≥ 50% proteome similarity boundary, the three groups within A2 identified by VIRIDIC are supported; (C) VICTOR-generated dendrogram of the proposed subclusters. Novel subclusters are indicated in red squares. The three A2 groups appear as monophyletic clades on the dendrogram, similar to how the other subclusters are represented, supporting the creation of two subclusters.

Interestingly, within the VIRIDIC alignment, subcluster A2 is “interrupted” by the A17 genome [Figure 3A]. This is unexpected as subcluster groups are based on “recognisable divisions” within nucleotide similarity alignments, so it would be anticipated that each subcluster would align together in a defined group. Therefore, this interruption would suggest that there is a discrepancy in the organisation of A2. Considering a nucleotide similarity threshold of ≥ 60%, there are three groups apparent within the VIRIDIC alignment of the A2 phages, which may be better represented by three subclusters [Figure 3A]. These groups are also reflected in the Gegenees proteome alignment of these phages when a proteome similarity threshold of ≥ 50% is applied [Figure 3B]. The VICTOR-generated phylogeny did in fact reveal that the current A2 subcluster is comprised of three monophyletic branches which support the groups identified in the previous alignments [Figure 3Ci]. This is quite unusual, as the other A subclusters included in the phylogeny have demonstrated that subclusters are monophyletic. Therefore, it is reasonable to propose the creation of two novel subclusters from two of the branches, with the largest branch remaining as A2, in order to implement as little reorganisation as possible [Figure 3Cii]. This would create a single subcluster for *Pukovnikviruses* and *Turbidoviruses* (A2), and the second subcluster would encompass the novel genera *Adzzyvirus*, *D29virus*, and *Serenityvirus*.

#### Cluster G - Supporting evidence for the removal of subcluster G5

**Figure 4:**
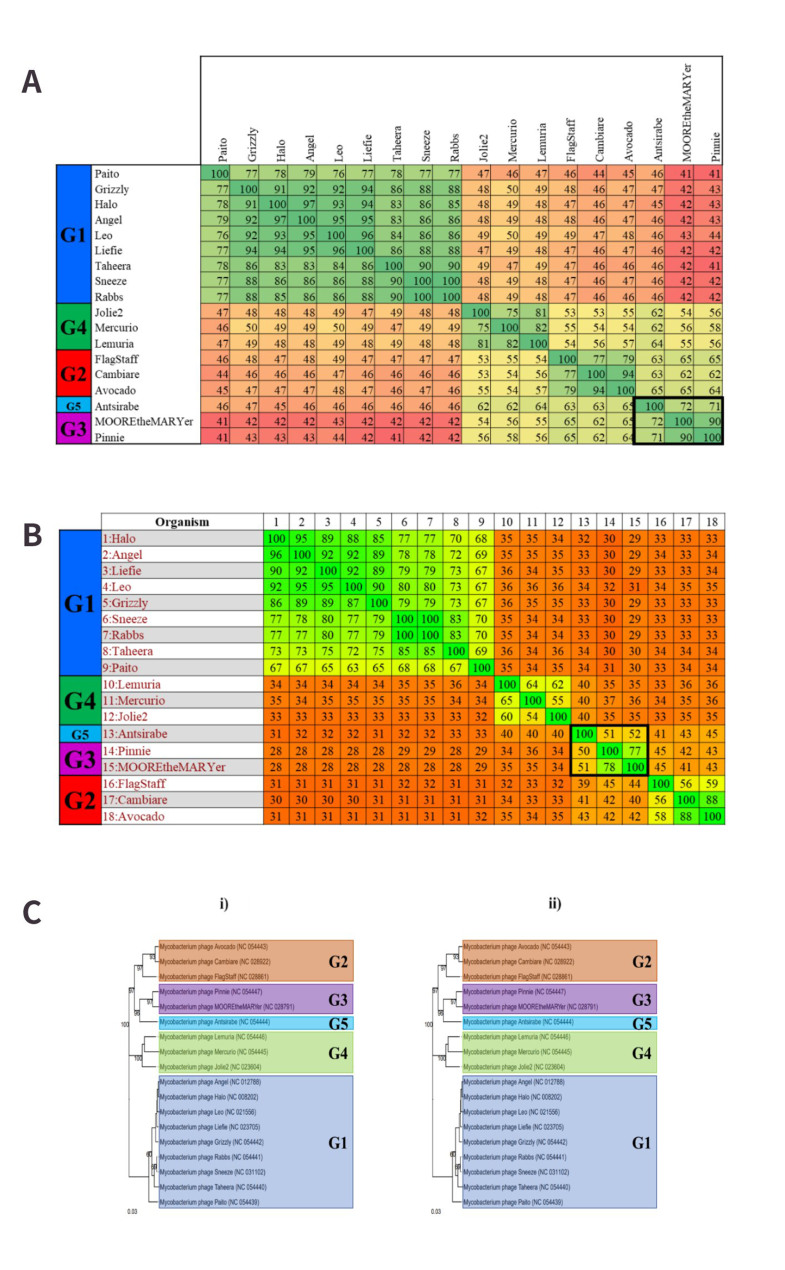
Cluster G analysis. Proposed groups are indicated in bold squares. (A) Subcluster and VIRIDIC-based genus assignments of cluster G. The nucleotide similarity values range from 41% to 100%. The single genus assigned to G3 and G5 is indicated by a bold square. The minimum threshold for genus inclusion is met; therefore, these three phages belong to a single group based on this analysis; (B) Gegenees proteomic analysis of the cluster G phages. The proteome similarity values range from 28% to 100%. A similarity boundary of ≥ 50% reflects the groups assigned by VIRIDIC; (C) VICTOR-generated phylogenetic tree of cluster G. (i) The existing subcluster assignments are indicated in coloured squares. It can be seen that the G3 and G5 phages appear to share a highly supported, monophyletic branch; (ii) The proposed changes to subcluster organisation. G3 has been expanded to include the single G5 genome, which better reflects the results of the VIRIDIC and Gegenees analyses.

Cluster G is a relatively small cluster featuring five subclusters and 18 genomes within this dataset. VIRIDIC did not identify any novel genera within this group, but rather assigned four genera to the five subclusters. Three of these genera reflected the G1, G2 and G4 subclusters (thereby supporting a relationship between genus and subcluster), whereas G3 and G5 were assigned a single genus based on the established ≥ 70% similarity demarcation [Figure 4A]. This contradicts the existing taxonomy of these latter subclusters, as the G5 MP, Antsirabe, belongs to its own genus, *Antsirabevirus* [Supplementary Table 1]. However, the Gegenees output for this cluster indicates that even at a protein level, a single genus for the G3 and G5 MP is supported based on the proposed ≥ 50% proteome similarity threshold [Figure 4B]. This threshold also supports the removal of Antsirabe from G5 and its inclusion within G3, as this similarity boundary has supported the novel subclusters proposed in previous sections [Figure 3]. Further support for assigning a single genus and a single subcluster to these two subclusters is provided by the VICTOR-generated phylogenetic tree, as the G3 and G5 are shown to share a singular branch, similar to the mono-phylogeny of the other genera and subclusters [Figure 4C].

#### Cluster H - One novel subcluster

**Figure 5:**
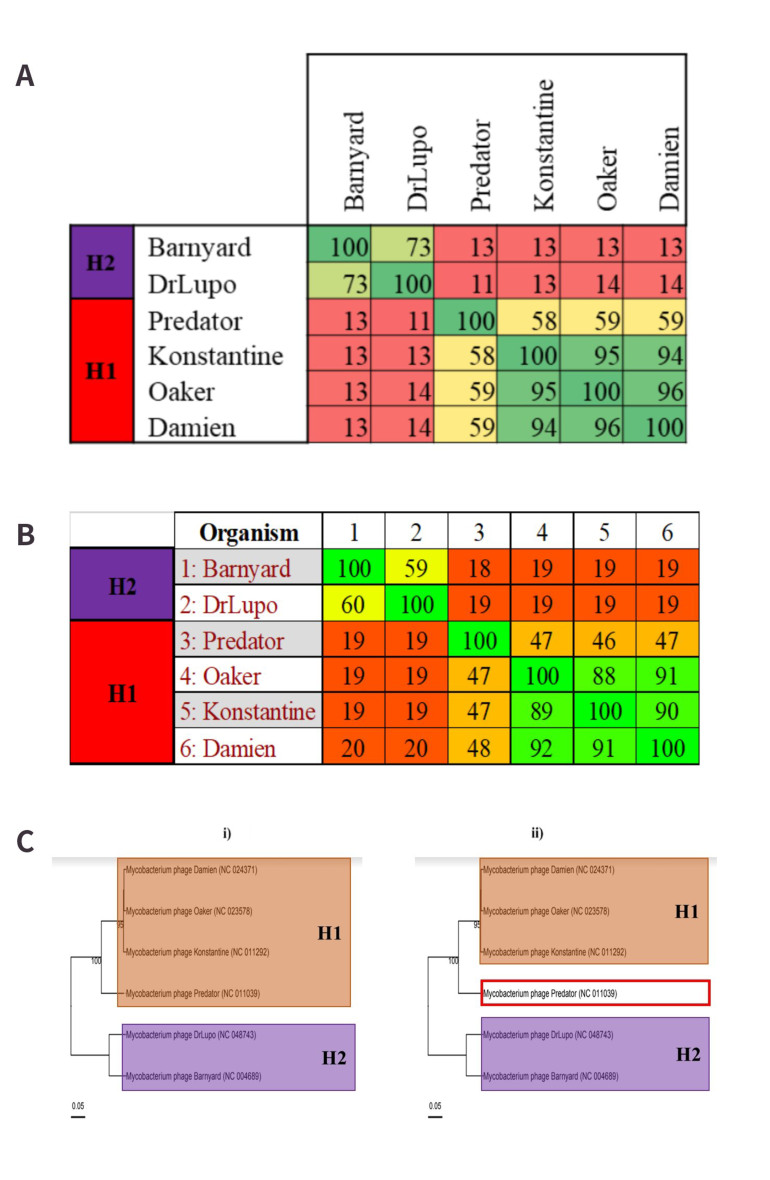
Proposed cluster assignments for cluster H. (A) VIRIDIC alignment of cluster H. The single *Predatorvirus*, Predator, does not meet the ≥ 70% nucleotide similarity threshold for inclusion with the larger H1 genus, *Konstantinevirus*; (B) Gegenees proteome alignment for cluster H. Predator also does not meet the proposed minimum proteome similarity threshold of 50% for inclusion in the same subcluster as any member of the *Konstantineviruses*; (C) (i) Existing subcluster classifications of cluster H. H1 and H2 are observable on two very distinct clades. Predator shares a clade with the other H1 viruses. (ii) Proposed subcluster creation for *Predatorviruses*. While Predator shares a clade with the *Konstantineviruses*, the divergence of its branch from the other phages would appear to reflect the < 50% proteome similarity noted in the Gegenees output, which would support the creation of a novel subcluster (indicated in the red square).

Within this dataset, cluster H is comprised of six phages that are organised into three genera and two subclusters. Subcluster H1 features two genera, *Predatorvirus* and *Konstantinevirus*. *Predatorvirus* is represented by a single MP, Predator. This phage does not meet the ≥ 70% nucleotide similarity required for inclusion with *Konstantinevirus* [Figure 5A]. The Gegenees analysis of this cluster also highlights that Predator does not meet the proposed ≥ 50% proteome similarity for inclusion with *Konstantinevirus*, which would further suggest that the *Predatorvirus* is a well-established genus [Figure 5B]. The phylogeny of this cluster features at least two very distinct branches that relate to subclusters H1 and H2 [Figure 5Ci]. Predator appears to be farther removed from the other H1 cohorts, and considering it fails to meet the proposed proteome threshold, there is therefore an argument in favour of creating a new subcluster for *Predatorvirus* [Figure 5Cii]. By creating this additional subcluster, the genus-subcluster hypothesis is reinforced further, as the three genera within cluster H will be assigned to three subclusters, respectively.

#### Cluster J - One novel genera and two novel subclusters

**Figure 6:**
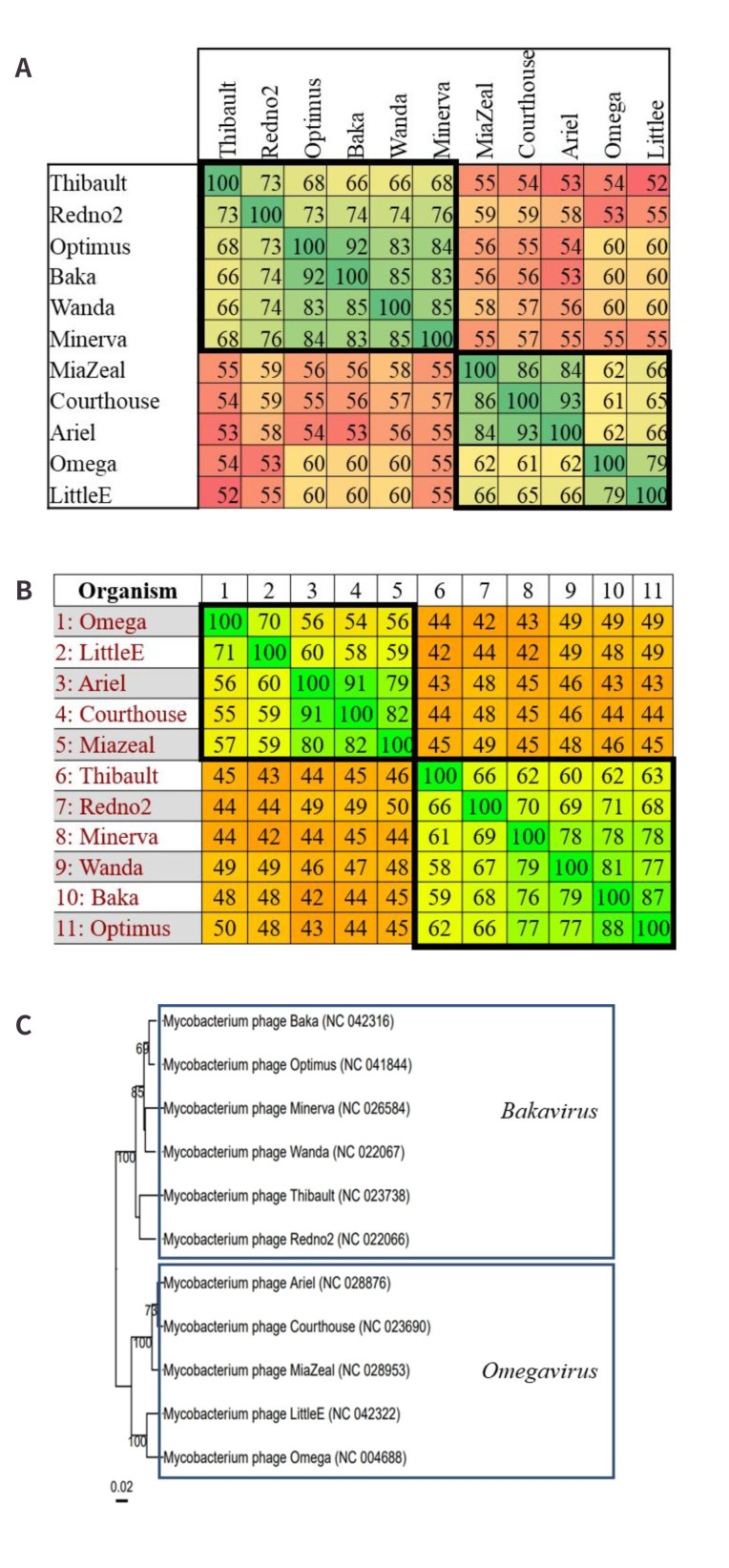
Novel genus assignments within cluster J. Novel groups are indicated within bold squares. (A) VIRIDIC alignment of the cluster J phages. Nucleotide similarity values range from 52% to 100%. When a similarity threshold of ≥ 60% is applied, two distinct groups can be observed in the alignment; (B) Gegenees proteome analysis of the cluster J phages. Proteome similarity values range from 42% to 100%. The two groups observed in the VIRIDIC analysis are reflected in this alignment when a threshold of ≥ 50% similarity is applied; (C) VICTOR-generated amino acid dendrogram. Two distinct branches are clearly depicted and therefore provide additional support for the creation of two genera and two subclusters within cluster J.

Currently, cluster J is not subdivided into subclusters. This would suggest a lack of “recognisable divisions” amongst the MP at a nucleotide level. However, VIRIDIC was able to allocate several genera to the 11 genomes included in the dataset from this cluster [Figure 6A], indicating there is at least a clear enough division to make taxonomic assignments based on nucleotide similarity. The VIRIDIC output for cluster J predicted 4 genera within this cluster based on the ≥ 70% similarity threshold [Figure 6A]. However, following visual inspection, it was noted that applying a slightly lesser threshold of ≥ 66% allows for a more noticeable distinction of two groups as opposed to four. The Gegenees output seems to agree with this, as applying the proposed genus, a threshold of ≥ 50% proteome similarity appears to reflect these two groups [Figure 6B]. As the VICTOR-generated dendrogram clearly illustrates two unique branches, it is arguable that the most confidence can be placed in the existence of two genera within this cluster. As it is a goal of this study to interfere with the existing taxonomic classifications as possible, it was decided to only acknowledge these two genera despite the deviation from the ≥ 70% threshold. The proposed nomenclature for these genera is *Bakavirus* and *Omegavirus*, as indicated in Figure 6C. *Omegavirus* is the established name of the existing genus recognised in cluster J, so only *Bakavirus* may be considered a novel finding.

With regards to potentially creating subclusters for these genomes, the creation of two subclusters within cluster J appears to be robustly supported, considering the VIRIDIC and Gegenees outputs highlight at least two groups, the latter of which can be supported by the proposed subcluster threshold of ≥ 50% proteome similarity. By creating a subcluster for each genus identified, i.e., J1 (*Bakavirus*) and J2 (*Omegavirus*), it would better reflect the diversity of these phages, and it would further support the proposed notion that genus assignment can be a predictor of subcluster formation and that ≥ 50% proteome similarity is a reliable threshold for subcluster creation.

#### Cluster K - Eight novel genera and nine novel subclusters

**Figure 7:**
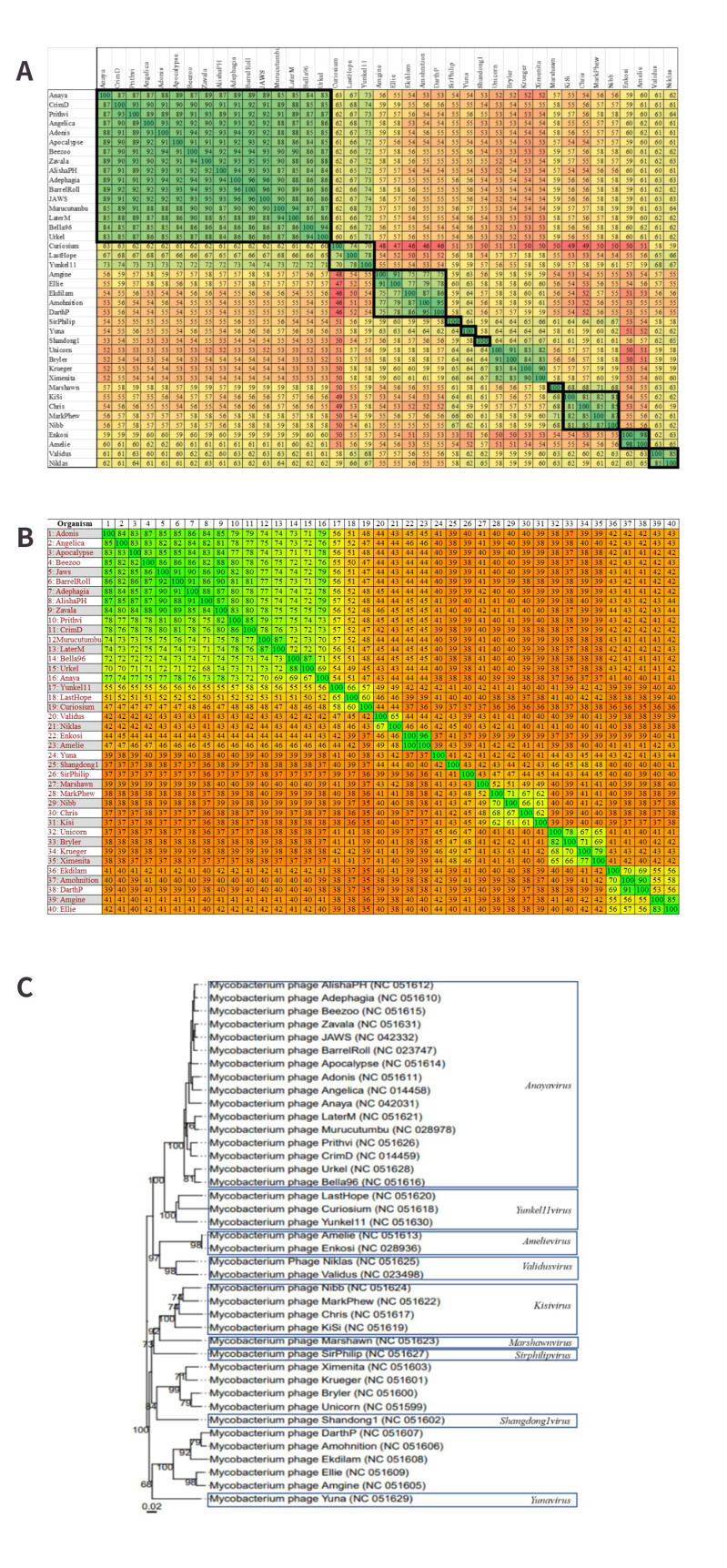
Novel groups are indicated in bold squares. (A) VIRIDIC-predicted genera of subcluster K1 and K6. The nucleotide similarity ranges from 46% to 100 %. Based on the genus demarcation of ≥ 70 nucleotide similarity, several novel groups can be identified; however, a few closely related groups do not have entirely clear distinctions between them; (B) Gegenees analysis of the K1 and K6 MP. The proteome similarity ranges from 35% to 100 %. This output largely supports the VIRIDIC-predicted genera, but using the established threshold of ≥ 50% does not completely clarify the boundaries between the proposed genera that appear closely related to their neighbours. Increasing the threshold to approximately 60% does little to improve this situation; (C) VICTOR-generated dendrogram of the proposed genera. This phylogenetic tree supports the creation of the novel genera proposed by VIRIDIC based on their monophyletic nature. *Anayavirus* was a previously existing genus that has now been reduced in size by the creation of the novel groups. Therefore, *Anayavirus* is not a novel finding.

In total, 66 cluster K phages were analysed to identify novel groups. Similarly to previous clusters, several K subclusters support the hypothesis that one subcluster can be assigned to a single genus, but there were two subclusters that disagreed with this observation. Based on the VIRIDIC analysis of this cluster, K1 and K6 appear to be comprised of several genera when considering the nucleotide similarity threshold of ≥ 70% [Figure 7A]. MP Yunkel11 and Marshawn appear to be closely related to the “neighbouring” genus, and visual inspection (similar to that applied for cluster J in the previous section) appears to support the combination of a few of the VIRIDIC-predicted genera based on these highly related groups. However, the subsequent Gegenees analysis of these phages [Figure 7B] provided clearer distinctions between these groups when a proteome similarity threshold of approximately 60% is applied. This 60% threshold also creates two groups out of the phages Ekdilam, Amohnition, DarthP, Amgine and Ellie, which VIRIDIC (and the existing ICTV/NCBI taxonomy) classifies as a single genus. While reducing the threshold to the previously adopted ≥ 50% recombines these two groups, it does not entirely clarify the boundaries between Yunkel11 and Marshawn and their neighbouring genera. The VICTOR analysis, on the other hand, was able to demonstrate the monophyletic nature of all the proposed genera, including that of Yunkel11 and Marshawn, indicating 8 novel groups, the names of which are listed in Figure 7C. The monophyletic natures of these genera mirror the monophyletic nature of the genera *Unicornvirus* and *Amginevirus*, which were included for comparative purposes.

**Figure 8:**
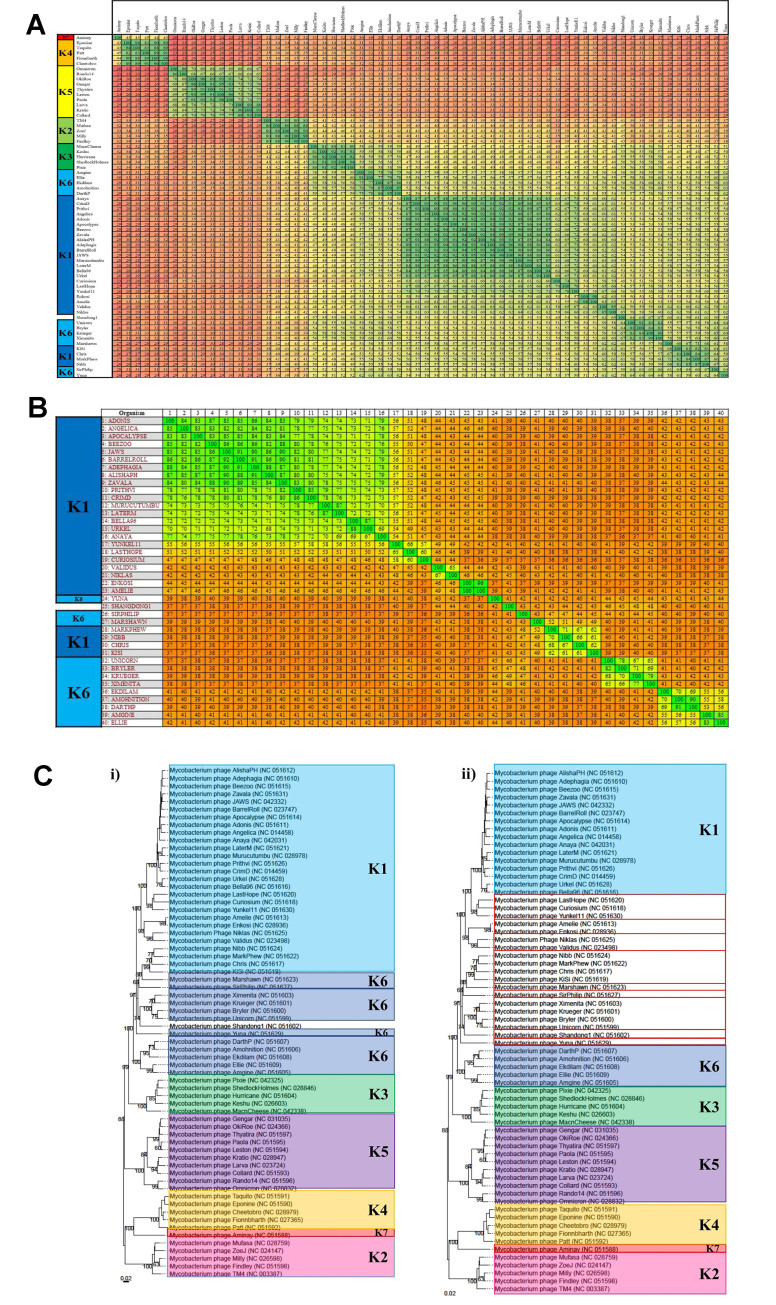
Subcluster analysis for cluster K. (A) VIRIDIC alignment of cluster K. The nucleotide similarity value range is 20% to 100%. Subclusters K1 and K6 appear particularly intertwined, as subgroups have formed from the original subclusters and are alternating in the alignment, suggesting clarification is required between the subclusters; (B) Gegenees analysis of the intertwined subclusters. The proteomic similarity value range indicated is 35% to 100%. Despite some rearrangement of K1 and K6, these subclusters are still intermingled. Applying a similarity threshold of approximately 50% appears to suggest that the creation of more subclusters would better describe the diversity of these phages; (C) VICTOR phylogeny of cluster K. (i) Existing organisation of the subclusters of cluster K. K6 appears to feature across four separate branches, only one of which appears to be truly monophyletic, which supports the VIRIDIC and Gegenees results; (ii) Proposed subclusters of cluster K. Novel clusters are indicated in red squares. K1 (light blue) has been reduced to include fewer phages and allow the creation of 4 novel subclusters. The smaller K6 branches have also been deemed novel subclusters. These groups better reflect the nucleotide and proteome studies and appear more visually similar to the other K subclusters indicated in the dendrogram.

Identifying monophyletic groups in the VICTOR analyses also proved extremely important for untangling subclusters K1 and K6 and redefining the boundaries of these groups. When the VIRIDIC output is compared to the subcluster assignments, it is clearly illustrated that K1 and K6 are intertwined with each other, while the remaining subclusters are quite distinct from each other [Figure 8A]. A similar (though not identical) intermingling is seen when the previous Gegenees alignment is compared with the subcluster assignments [Figure 8B]. As established for previous clusters, it appears that approximately 50% proteome similarity is quite a robust metric for the identification of subclusters. Therefore, the creation of several novel subcluster assignments would likely better reflect the genetic diversity of these MP highlighted in the VIRIDIC and Gegenees outputs. Based on the VICTOR analysis and the observation that subclusters form monophyletic groups, it appears that there is sufficient support to warrant the addition of nine novel subclusters in total [Figure 8C]. These additional subclusters would support the hypothesis that one genus can be assigned to a single subcluster, which in this case helps reflect the diversity of the genera that only feature one MP within this dataset.

## DISCUSSION

Phages will most likely prove an essential part of the effort to overcome the very concerning threat of antibiotic resistance. As the most abundant biological entities on Earth^[29]^, an overwhelming arsenal is hypothetically available for the design of phage-based therapeutics and diagnostics, and the possibility to genetically engineer the phages makes the composition of phage cocktails endless. In order to capitalise upon the diversity of phages, it is important to have robust classification systems in place. Lawrence *et al.* described in immense and commendable detail the difficulties of applying traditional Linnaean classification (essentially traditional hierarchal taxonomy based on shared characteristics) with particular regard for how this style of classification underrepresents the diversity of phages, especially when genetic mosaicism is considered^[7]^. Many of the concerns raised by Lawrence *et al.*^[7]^ have been satisfied by the roadmap for genome-based taxonomy proposed by Turner *et al*.^[12]^. The roadmap recommends the abolishment of many of the Linnaean-type classifications and recommendation that whole genomes (as opposed to a core genome) be considered when assigning groups. VIRIDIC^[10]^ (which predicts genus and species assignments based on pairwise nucleotide comparisons with consideration for genome length and aligned genome fraction) has been heavily employed in a massive undertaking to update the taxonomy of phages according to the Turner *et al.* (2021) proposal (ICTV Master Species List; https://talk.ictvonline.org/files/master-species-lists/m/msl/12314). For this reason, VIRIDIC was selected as the initial analysis to identify groups of MP that may belong to novel genera.

After the identification of potentially novel genera in this study by comparing the VIRIDIC results to the existing taxonomic information, the genomes belonging to the predicted genera were analysed with Gegenees^[21]^. The reasoning for performing this analysis is that (hypothetically) the protein-based alignments would reflect the VIRIDIC (i.e., nucleotide) alignments and provide additional support and further confirm the predicted genera (although it should be noted that the genetic mosaicism, temperate lifestyle and limited host range of MP often makes nucleotide and proteomic analyses complicated; ^[24]^). If supported by both the VIRIDIC and Gegenees outputs, phylogenetic trees were expected to illustrate the novel genera as monophyletic branches^[12]^. Following this workflow, 20 well-supported novel genera were identified across three clusters, A, J and K. In one instance, evidence supporting the abolition of a genus in cluster G (*Antsirabevirus*) was presented. These genera were mostly identified following the widely accepted ≥ 70% nucleotide similarity demarcation proposed by Turner *et al.* which was heavily employed in the recently ratified taxonomy^[12]^. Typically this was supported by the Gegenees output as the application of a ~ 50% proteome similarity threshold clarified the boundaries between genera, which was the case of cluster J. For this cluster, a nucleotide similarity threshold of ≥ 66% was noted to be the minimum similarity value required for genus inclusion when the Gegenees and VICTOR outputs were considered in parallel. While the decision to change the threshold may seem arbitrary, the analyses presented in this study demonstrate that ≥ 50% proteome similarity and monophyletic groups are also strong indicators of genus groups and can be considered robust validations of genus assignments. It would therefore be recommended that this threshold of ~ 50% proteome similarity be applied to Gegenees analyses as additional supporting evidence for VIRIDIC predictions, which will allow researchers the opportunity to identify the most robust groups in circumstances where many nucleotide similarity values are approaching but not equal to 70%. Similarly, the VIRIDIC/Gegenees proposed genera should demonstrate a monophyletic nature (which proved especially important for clarifying the boundaries between the proposed novel genera within cluster K) [Figures 7 and 8].

As the taxonomic information regarding phages now reflects greater diversity than previously thought, it is decided to also explore the Actinobacteriophage database cluster and subcluster assignments to determine if they reflected the diversity described by the ICTV/NCBI taxonomy. It is pertinent that this classification system is reliable, as there have been studies designed with the understanding that MP belonging to the same subcluster may have similar attributes like codon usage bias or host range (e.g., ^[30,31]^) and approximated groups could lead to hypotheses that falsely include or exclude phages. Such hypotheses may therefore mischaracterise the molecular and/or phenotypic functionality of MP, which could have a knock-on effect on the design of phage-based therapies and diagnostics. While it is largely accepted that the current MP clusters are assigned based on the most convenient groups, as opposed to genetic or evolutionary accuracy^[6,18,20]^, importantly, it has been previously noted that organisation of MP into appropriate clusters/subclusters has become difficult as the number of available sequences increases^[18]^. Regarding subcluster classifications, there is currently no formal demarcation of “subcluster” and subcluster assignments are based on “recognisable divisions” in nucleotide similarity, as addressed in the Introduction^[18]^. It was noted during the initial curation of taxonomic and cluster information for the 721 MP genomes that there was a potential link between genus and subcluster assignments (Figure 1; the genus-subcluster hypothesis), i.e., that each subcluster comprised a single genus. This link appeared sound following the comparison of the existing taxonomy and the novel genera proposed in this study.

In some cases, like in cluster A, novel genera were assigned to a single subcluster, which further supports the suggestion that a single genus can be assigned to each subcluster. In other cases, like cluster J which currently lacks subclusters, the creation of genera appeared to warrant the creation of subclusters based on the “recognisable divisions” within the nucleotide alignments^[19]^ that were subsequently supported by the Gegenees and VICTOR analyses. Notably, clusters A and K featured inconsistent alignment of subclusters and VIRIDIC highlighted distinct groups that would suggest smaller subclusters could be formed from larger ones. The Gegenees analyses broadly paralleled the VIRIDIC outputs and supported reorganising the subclusters into smaller groups; however, it was quite difficult to clearly and distinctly define the groups within cluster K as described above and more consideration was given to the VICTOR output in that case. Interestingly, the Gegenees threshold (broadly speaking, cluster K appears to be an exception) for subcluster classifications appeared to be ~ 50% proteome similarity, which is the threshold proposed for genus identification within Gegenees outputs presented in this study. This creates additional reassurance in the genus-subcluster relationship hypothesis, as the threshold for genus and subcluster recognition is the same. Finally, the VICTOR-generated phylogeny illustrated all proposed subclusters as monophyletic, even the minority of those comprised of more than one genus. In total, 13 novel subclusters were proposed and the single phage presenting the subcluster G5 was recommended for inclusion in subcluster G3 as it met the VIRIDIC (≥ 70%), Gegenees (~ 50%) and VICTOR (monophyletic branch) parameters for inclusion in subcluster G3. Overall, it appears as though the criteria for genus inclusion are adequate to support the creation (or abolition) of subclusters, thereby formalising this classification.

While the 721 MP selected for this study are a small cohort of the more than 12,000 isolated MP, they represent approximately one-third of sequenced MP, which is a sizeable sample size. Although the genus-subcluster link is not infallible - and the limitations of DNA- and proteome-based comparisons along with genetic mosaicism which has been briefly discussed should not be completely disregarded, novel genus assignment appears to be a reliable indicator of subcluster creation. The original 83.3% of the dataset that supported the hypothesis increased to 97.6% when the 20 novel genera and 13 novel subclusters identified in this study were considered. Overall, these results highlight the necessity to frequently revise taxonomic classifications (potentially as a routine feature of novel phage genome characterisation) and ensure the fidelity of cluster and subcluster assignments as phage taxonomy evolves and more of the viral biosphere is characterised. By recognising and maintaining the genus-subcluster relationship between the taxonomic and clustering classification systems as much as possible, it will ensure that the diversity of MP is accurately reflected in both systems as more MP are sequenced and novel MP are isolated. Robust and linked classification systems could then aid rapid phage selection for research, therapeutic and diagnostic purposes as closely related phage will be easily defined within a cluster.

## DECLARATIONS

### Availability of data and materials

The original taxonomy and cluster/subcluster assignments of the 721 mycobacteriophages included in the dataset and the changes proposed in this study are available in Supplementary Table 1.

### Copyright

© The Author(s) 2023.
